# Optimal timing of GnRH antagonist initiation in IVF-ET: a retrospective cohort study on advanced maternal age women

**DOI:** 10.3389/fendo.2024.1340230

**Published:** 2024-02-05

**Authors:** Qiao-Song Han, Yue Zhou, Ying Xu, Kai-Liang Ai, Jing-Yan Song, Zhen-Gao Sun

**Affiliations:** ^1^ The First Clinical College, Shandong University of Traditional Chinese Medicine, Jinan, China; ^2^ The College of Traditional Chinese Medicine, Shandong University of Traditional Chinese Medicine, Jinan, Shandong, China; ^3^ Reproductive Center of Integrated Medicine, The Affiliated Hospital of Shandong University of Traditional Chinese Medicine, Jinan, China

**Keywords:** advanced maternal age, cumulative live birth rate, Cox proportional hazard model, fixed GnRH-ant protocol, flexible GnRH-ant protocol

## Abstract

**Background:**

Several studies have compared the effects of fixed and flexible gonadotropin releasing hormone antagonist (GnRH-ant) protocols during *in vitro* fertilization and embryo transfer (IVF-ET). However, which GnRH-ant initiation strategy is better remains controversial. Moreover, no studies have assessed the optimal timing of GnRH-ant initiation in women of advanced maternal age (AMA).

**Methods:**

In this retrospective cohort study, a total of 472 infertile women aged ≥ 35 years old undergoing their first IVF cycle from August 2015 to September 2021 at a tertiary academic medical center were recruited, of whom 136 followed fixed GnRH-ant protocol and 336 followed flexible GnRH-ant protocol. The primary outcomes measured were the cumulative live birth rate (CLBR) per IVF cycle and the time to live birth (TTLB) from the date of oocyte retrieval. Cox proportional models were used to calculate the hazard ratio (HR) and 95% confidence interval (CI) of CLBR regarding GnRH-ant timing.

**Results:**

No significant difference in CLBR was found between the fixed and flexible GnRH-ant groups (27.9% vs 20.5%, p=0.105). The TTLB was also comparable between groups (10.56 vs 10.30 months, p=0.782). The Kaplan-Meier analysis for CLBR also showed comparable results between groups (P=0.351, HR=0.83; 95%CI: 0.56-1.23). After establishing a multiple Cox proportional hazard model, the fixed GnRH-ant group still had comparable CLBR with the flexible GnRH-ant group (HR=0.85; 95%CI: 0.53-1.38; P=0.518). Subgroup and sensitivity analyses also demonstrated similar results.

**Conclusion:**

GnRH-ant protocols can be tailored to the needs of AMA women, and timing of GnRH-ant initiation can be adjusted flexibly.

## Introduction

Nowadays, GnRH-ant protocol has become increasingly widely used clinically to prevent the premature rise in luteinizing hormone (LH) during controlled ovarian stimulation (COS) before IVF-ET (1, 2). In general, there are two main protocols for GnRH-ant initiation, namely fixed and flexible protocols (3). In the fixed GnRH-ant protocol, irrespective of the size of follicles, the GnRH-ant is initiated every day on a fixed day of COS. Likewise, in the flexible GnRH-ant protocol, GnRH-ant is initiated based on the size of the follicle and/or the level of serum hormones, such as estradiol (E_2_) and luteinized hormone (LH).

In COS, changing LH levels is likely to be the most important factor that influences GnRH-ant initiation at different times. A reproductive hormone secreted by the pituitary gland, LH is necessary for the biosynthesis of steroids, folliculogenesis, and ovulation (4). The hypothesis called “LH window” emphasized that a “threshold” and a “ceiling” formed an optimal range for serum LH level during COS. Both excessive and insufficient LH may exert a negative effect on cycle outcomes (5).

Women at AMA are a population with expected poor IVF-ET outcomes, and none of the current treatments or strategies showed truly uplifting results (6, 7). According to previous studies, in addition to ovarian aging, AMA women also have the characteristics of a “relative LH deficiency” and are more prone to a “premature LH surge”, hence the dissatisfying outcomes (8, 9). In light of these findings, the question arises: when is the optimal time to initiate GnRH-ant in AMA women in order to stabilize serum LH levels within a reasonable range and to optimize IVF-ET outcomes?

In a recent systematic review, different GnRH-ant initiation protocols were compared, and a fixed protocol was found to be significantly superior to a flexible protocol in terms of ongoing pregnancy rates (10). Study populations in the included studies were heterogeneous, and further high-quality studies need to be conducted with more homogeneous cohorts to confirm the findings. As a result, the purpose of this study is to compare the efficacy of two GnRH-ant protocols among AMA women in terms of CLBR and other pregnancy outcomes during one complete IVF cycle, including fresh embryo transfers and all subsequent FETs.

## Materials and methods

The study was reviewed and approved by the Ethics Committee of the affiliated hospital of Shandong University of Traditional Chinese Medicine (SDUTCM). The ethics committees waived the requirement for informed consent due to the retrospective nature and anonymous data of this study. The study is performed completely in accordance with the Declaration of Helsinki and its further amendments (11).

### Study design and population

The study included AMA women aged ≥ 35 years old, who underwent the first IVF-ET treatment from August 2015 to September 2021 at a tertiary academic medical center. All included women received GnRH-ant protocol for ovarian stimulation, and the initiation time of GnRH-ant is based on standard operating procedure (SOP) in our center: (1) Fixed GnRH-ant protocol: GnRH-ant was initiated at day 6 of stimulation; (2) Flexible protocol: GnRH-ant was initiated when the largest ovarian follicle has reached a diameter of 12 to 14 mm. The definition of GnRH-ant protocol in accordance with most of the previous studies (10). The study follow-up period was two years from the day of oocyte retrieval. Each woman was included only once in the analysis. Our exclusion criteria were as follows: (1) Preimplantation genetic testing or diagnosis cycles; (2) Cycles with cryopreserved oocytes and/or donor oocytes; (3) Women with a history of ovarian surgery, genital tumors, endometriosis, or uterine malformations; (4) Women with a history of recurrent spontaneous abortion (≥ 3 times). Given the study objective, no upper age limit was set for the included women.

### Ovarian stimulation protocol

Eligible participants received ovarian stimulation with a GnRH-ant protocol from menstrual cycle days 1–3. The starting dosage of recombinant follicle-stimulating hormone (r-FSH) (Gonal F, Merck Serono S.p.A, Modugno, Italy) was 150-300 IU per day. The starting dosage of gonadotrophin was determined based on individual patient baseline characteristics, such as age and BMI. The follow-up dosage was adjusted according to the ovarian response, follicular growth monitored by transvaginal ultrasonography and serum sexual hormone concentrations. Based on the personal experience and discretion of physicians, a fixed or flexible GnRH-ant protocol was performed using 0.25mg daily GnRH-ant (cetrorelix acetate, Cetrotide; Merck Serono, Germany) from Day 6 of stimulation or as soon as the diameter of the leading follicle reached 12-14 mm. When two or more follicles measured 18 mm or more, Ovidrel 250 μg (Merck Serono S.p.A., Modugno, Italy) was given to trigger the final maturation of the oocytes. Oocyte retrieval was performed approximately 35-37 hours later.

### IVF and embryo evaluation

Retrieved oocytes were inseminated by IVF, unless intracytoplasmic sperm injection (ICSI) was indicated in case the total number of progressive motile sperm count was < 5×10^6^ or the normal morphology of sperm was < 1%. The cleavage embryos were defined as high-quality with no less than six blastomeres and ≤ 20% fragmentation. Blastocysts with a Gardner score of 3BB or higher is high-quality and available for FET.

### Embryo transfer and luteal support

A maximum of two embryos were transferred on day 3 after oocyte retrieval. Surplus embryos or blastocysts of good quality were cryopreserved utilizing the previously described fast-freezing procedure (12). Freeze-all strategy was applied in patients with elevated serum progesterone >1.5 ng/ml during COS and those with an insufficient endometrial thickness (<7 mm). The FET regimen was performed in natural or modified natural cycles for ovulatory women and standard hormone replacement cycles for anovulatory women. For all patients undergoing embryo transfer, LPS will continue until 10-12 weeks of gestation, with an intramuscular progesterone injection of 40mg per day, plus dydrogesterone tablet (Duphaston, Abbott, Hoofddorp, Netherlands) 30mg per day or vaginal progesterone (8% Crinone, Merck Serono, Switzerland) 90mg per day. The LPS will be discontinued if the serum pregnancy test is negative or the transvaginal ultrasound reveals pregnancy failure.

### Outcome measurements

The primary outcome was the CLBR per IVF cycle. Live birth was defined as any birth event in which at least one infant (>28 weeks of gestation) was born alive. A cumulative live birth was defined as the first live birth achieved in either an ET or a FET cycle. We chose a conservative approach to calculate the CLBR, which means if all embryos were used up in an IVF cycle, or a live birth was not achieved within 2 years from the day of oocyte retrieval, we considered the woman did not achieve a cumulative live birth. The co-primary outcome was the time to live birth (TTLB), which was measured as the interval between the date of the oocyte retrieval and the date of delivery, expressed in number of months. Secondary outcomes included total gonadotrophin dose, the incidence of premature LH surge (LH level >10 IU/L) and ovulation, sex hormone profile at hCG trigger day, number of oocytes retrieved, number of 2PN zygotes, number of transferred embryos, clinical pregnancy rate, early pregnancy loss rate, and live birth rate. Clinical pregnancy was confirmed when one or more gestational sacs were detected on transvaginal ultrasound scanning. Early pregnancy loss was defined as the spontaneous loss of a diagnosed clinical pregnancy before 12 weeks gestation.

### Statistical analysis

Continuous variables were expressed as median or interquartile range [25^th^, 75^th^] according to the distribution of the variables, whereas categorical variables were presented as frequency (n) and percentage (%). The distribution of normality was tested by histogram, Q-Q plot, and Shapiro Wilk test. Student t-test, Mann-Whitney U test, or chi-squared test will be used to test for differences as appropriate. Kaplan-Meier curves were applied for the cumulative live birth events for the fixed GnRH-ant and flexible GnRH-ant groups and were compared using the log-rank test. Censor was applied when a woman had not achieved a live birth within 2 years, had no surplus available embryos, or withdrew for any reason. The hazard ratio (HR) and 95% confidence interval (CI) of CLBR were calculated from univariate and multivariate Cox proportional models in terms of the effect of the timing of GnRH-ant initiation. Multi-model 1 was adjusted for age, body mass index (BMI), duration of infertility, type of infertility, basal FSH level, basal LH level, antral follicle count, and Infertility indicators. Model 2 was adjusted for all variables in Model 1, with multiple imputations to missing values. Sensitivity analysis was conducted in subpopulations with fresh embryo transfer and those with freezing-all cycles. Another subgroup analysis included a comparison in women aged ≥ 40 years. Besides, an additional analysis was also performed using propensity score matching to compare the primary outcomes. All statistical analysis were performed using R software version 4.3.1(R Core Team, Vienna, Austria). The differences were considered statistically significant when the P-value was <0.05.

## Results

**Figure 1:**
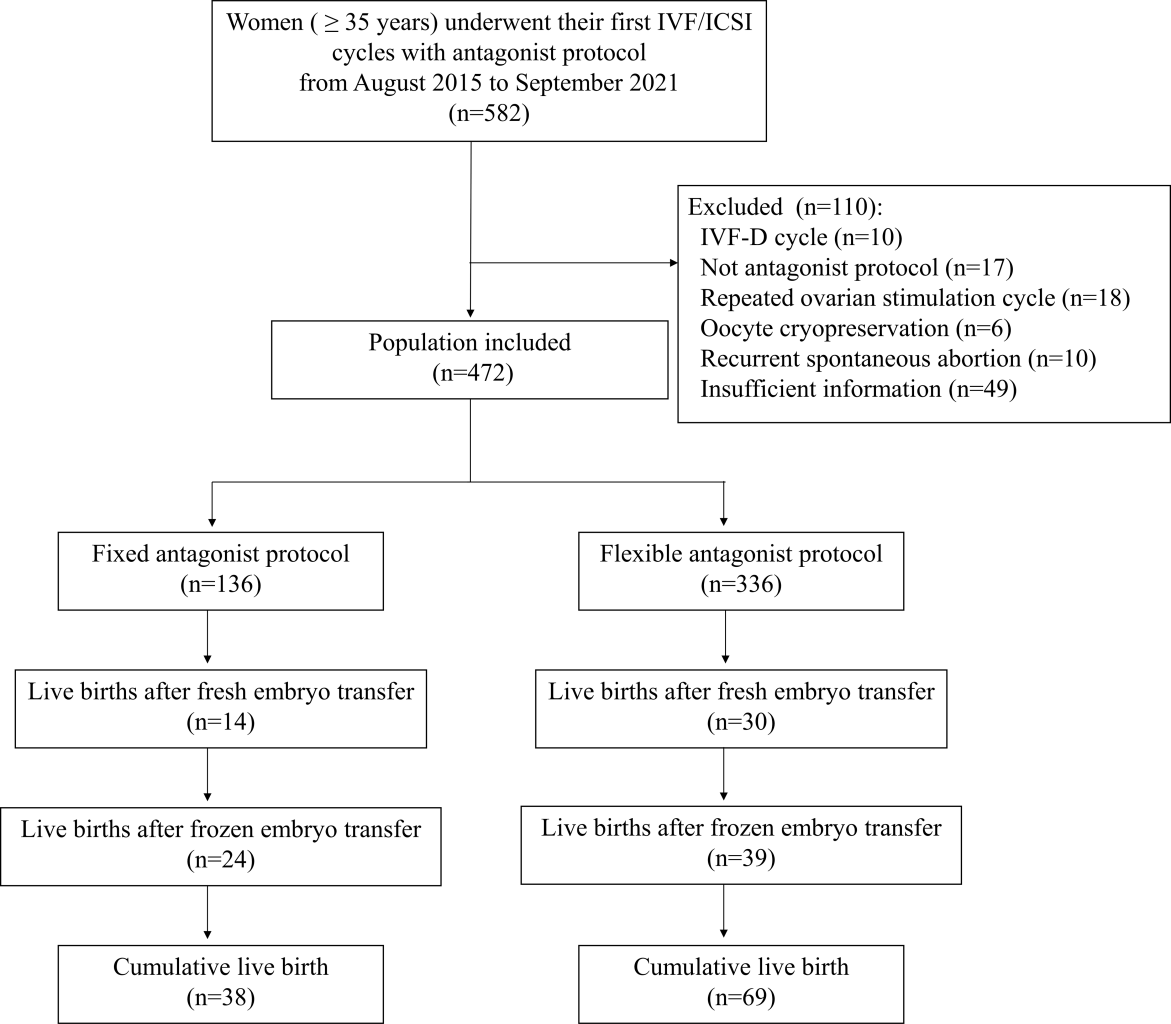
Flow chart of the study. IVF, *in vitro* fertilization; ICSI, Intracytoplasmic sperm injection; IVF-D, *in vitro* fertilization with donor sperm.

A total of 582 women were screened for eligibility, of which 472 women were included for analysis (136 with fixed GnRH-ant protocol and 336 with flexible GnRH-ant protocol). A total of 107 women with at least one live birth within 2 years were observed in this study (**Figure 1**).

### Baseline patient characteristics

The baseline characteristics are presented in **Table 1**. The patient characteristics were comparable between the two groups, as no significant difference was found in the age, BMI, duration of infertility, type of infertility, basal FSH level, basal LH level, antral follicle count, and infertility indicators. For both groups, the median age was 38 years old, a larger proportion of women had secondary infertility, and the female factor was the major indication for infertility.

**Table 1:** Baseline characteristics of women in two groups.

Characteristics	Fixed GnRH-ant group	Flexible GnRH-ant group	P value
Patients	136	336	
Age (years)	38.00 [36.00, 41.00]	38.00 [36.00, 41.00]	0.936
BMI (kg/m^2^)	23.20 [21.20, 25.00]	23.65 [21.65, 26.00]	0.059
Duration of infertility	3.00 [1.00, 5.00]	3.00 [1.00, 5.00]	0.757
Type of infertility			1.000
Primary infertility	29 (21.3)	71 (21.1)	
Secondary infertility	107 (78.7)	265 (78.9)	
Basal FSH level (IU/L)	7.91 [6.52, 9.81]	7.72 [6.32, 9.73]	0.658
Basal LH level (IU/L)	4.18 [3.46, 5.99]	4.26 [3.09, 5.61]	0.234
Antral follicle count	16.00 [10.50, 22.50]	15.00 [10.00, 22.00]	0.367
Infertility indicators			0.685
Female factor	115 (84.6)	273 (81.2)	
Male factor	3 (2.2)	8 (2.4)	
Mixed factor	18 (13.2)	55 (16.4)	

Continuous data are represented as median (25th and 75th percentile) because of nonnormal distribution, and categorical variables are represented as number (%).

GnRH-ant, gonadotropin releasing hormone antagonist; BMI, body mass index; FSH, follicle-stimulating hormone; LH, luteinizing-hormone.

### Ovarian stimulation and pregnancy outcomes

For the timing of GnRH-ant initiation in the flexible group, a larger proportion of women (58.3%) initiated the GnRH-ant after day 6 of the COS (**Table 2**). The risk of premature LH surge was similar between groups (23.1% vs. 25.2%, P=0.730). Both groups showed comparable results in terms of the hormone profile at hCG trigger day, number of retrieved oocytes, number of 2PN zygotes, and number of transferred embryos. Fresh embryo transfers were performed more frequently in women undergoing flexible GnRH-ant protocol (38.2% vs. 45.2%), while more women in the fixed group received the freeze-all strategy (49.3% vs. 38.1%). However, the difference was not statistically significant (P=0.078). The embryo transfer and pregnancy outcomes are shown in **Table 3**. In the fixed GnRH-ant group, 136 women underwent 165 transfer cycles, including 52 fresh ET and 113 FET cycles, whereas 405 transfer cycles (152 fresh ET and 253 FET cycles) were undertaken by 336 women in the flexible GnRH-ant group. No significant difference was identified in the clinical pregnancy rate (38.5% vs 30.5%), live birth rate (26.9% vs 19.7%), and early pregnancy loss rate (30% vs 32.6%) in fresh ET cycles (all P > 0.05). Similarly, results did not differ in FET cycles.

**Table 2:** Ovarian stimulation outcomes.

	Fixed GnRH-ant group	Flexible GnRH-ant group	P value
Patients (n)	136	336	
Timing of antagonist administration			<0.001
Day 6 of ovarian stimulation	136 (100.0)	0 (0.0)	
> Day 6 of ovarian stimulation	0 (0.0)	196 (58.3)	
< Day 6 of ovarian stimulation	0 (0.0)	140 (41.7)	
Total Gn dose (IU)	2250.00 [1800.00, 2856.25]	2325.00 [1800.00, 3000.00]	0.955
Premature LH surge	30 (23.1)	83 (25.2)	0.730
Premature ovulation	2	3	
Hormone profile at hCG trigger day			
E_2_ level (pg/ml)	2409.00 [1238.07, 3821.00]	1979.50 [1121.36, 3771.75]	0.313
LH level (IU/L)	3.04 [1.98, 5.59]	2.86 [1.82, 4.97]	0.366
P level (ng/ml)	0.96 [0.64, 1.48]	0.93 [0.60, 1.41]	0.371
No. of oocytes retrieved	7.00 [4.00, 11.00]	8.00 [4.00, 11.00]	0.819
Insemination method			0.561
IVF	106 (80.3)	246 (75.9)	
ICSI	23 (17.4)	71 (21.9)	
IVF+ICSI	3 (2.3)	7 (2.2)	
No. of 2PN zygotes	4.00 [2.00, 7.25]	4.00 [2.00, 7.00]	0.986
No. of transferred embryos	2.00 [1.00, 4.00]	2.00 [1.00, 4.00]	0.485
Cycle outcomes			0.078
Cycle with fresh embryo transfer	52 (38.2)	152 (45.2)	
Cycle with freeze-all strategy	67 (49.3)	128 (38.1)	
Cycle cancellation due to no available embryo	17 (12.5)	56 (16.7)	

Continuous data are represented as median (25th and 75th percentile) because of nonnormal distribution, and categorical variables are represented as number (%).

GnRH-ant, gonadotropin releasing hormone antagonist; Gn, Gonadotrophin; LH, Luteinizing hormone; hCG, Human chorionic gonadotropin; E2, estradiol; P, progesterone; IVF, In vitro fertilization; ICSI, Intracytoplasmic sperm injection; CLBR, cumulative live birth rate; TTLB, Time to first live birth.

**Table 3:** Embryo transfer and pregnancy outcomes.

	Fixed GnRH-ant group	Flexible GnRH-ant group	P value
Patients (n)	136	336	
Total no. of transfer cycles	165	405	
Cycle type of embryo transferred			0.174
Fresh ET	52 (31.5)	152 (37.5)	
FET	113 (68.5)	253 (62.5)	
Fresh cycle outcomes			
Clinical pregnancy rate	20 (38.5)	46 (30.3)	0.358
Live birth rate	14 (26.9)	30 (19.7)	0.372
Early pregnancy loss rate	6 (30.0)	15 (32.6)	1.000
FET outcomes			
Clinical pregnancy rate	34 (30.1)	60 (23.7)	0.197
Live birth rate	24 (21.2)	39 (15.4)	0.173
Early pregnancy loss rate	10 (29.4)	15 (26.8)	0.787
CLBR	38 (27.9)	69 (20.5)	0.105
Time to first live birth (months)	10.56 [8.73, 12.80]	10.30 [8.67, 13.23]	0.782

Continuous data are represented as median (25th and 75th percentile) because of nonnormal distribution, and categorical variables are represented as number (%).

GnRH-ant, gonadotropin releasing hormone antagonist; FET, frozen-thawed embryo transfer; CLBR, cumulative live birth rate.

### Cumulative live birth rate and time to live birth

**Figure 2:**
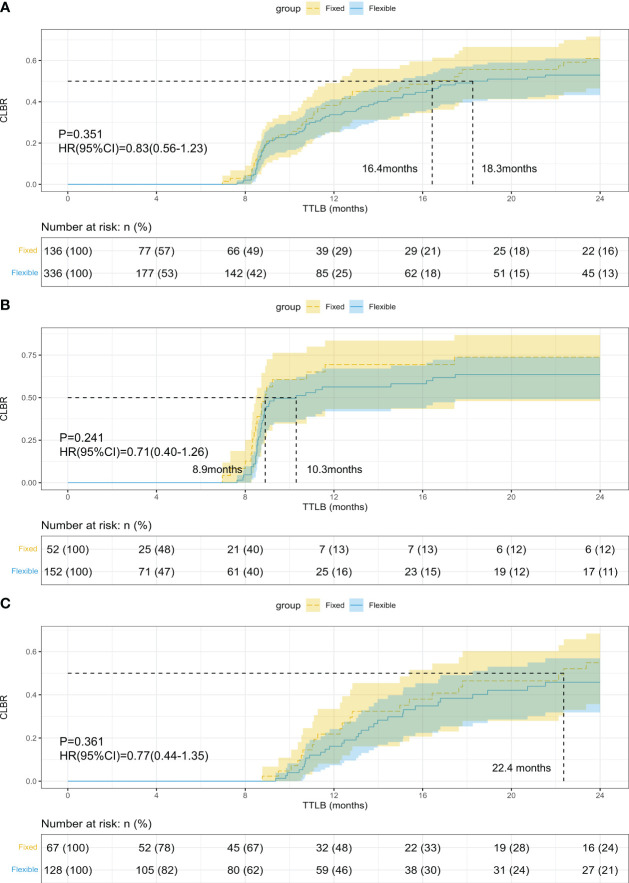
Kaplan-Meier curves of the cumulative live birth rate (CLBR) in women who underwent fixed and flexible gonadotropin releasing hormone antagonist protocol. **(A)** CLBR of all the patients; **(B)** CLBR in women with fresh embryo transfer; **(C)** CLBR in women freezing all embryos.

The CLBR was comparable between groups (27.9% vs 20.5%, p=0.105), and the TTLB was similar in the fixed and flexible GnRH-ant groups (10.56 vs 10.30 months, p=0.782, shown in **Table 3**). **Figure 2** presents the results of the Kaplan-Meier analysis for CLBR. In the total population, the median time to live birth was 16.4 months and 18.3 months, respectively. The results of the log-rank test showed no significant difference in CLBR (P=0.351, HR=0.83; 95%CI: 0.56-1.23, **Figure 2A**). After stratified with the embryo transfer protocol, the results did not differ in women with fresh embryo transfer (P=0.241, HR=0.71; 95%CI: 0.40-1.26, **Figure 2B**) and those with freezing-all cycles (P=0.361, HR=0.77; 95%CI: 0.44-1.35, **Figure 2C**).

We also established Cox proportional hazard models to determine the effect on CLBR according to the fixed or flexible GnRH-ant protocol (**Table 4**). In model 1, after adjusting for age, BMI, duration of infertility, type of infertility, basal FSH level, basal LH level, antral follicle count, and infertility indicators, women who underwent fixed GnRH-ant protocol had a similar CLBR compared with those receiving flexible GnRH-ant protocol (HR= 0.85; 95% CI: 0.53-1.38; P=0.518). Model 2 was adjusted for all variables in model 1 with the addition of multiple imputations to missing values, in which the results were still robust (HR= 0.86; 95% CI: 0.57- 1.30; P=0.477). For sensitivity analysis, the Cox models 1 and 2 were performed in subgroups divided by embryo transfer strategy (**Table 4**). In women with fresh ET (model 1: HR= 0.65; 95% CI: 0.31-1.35; P=0.250; model 2: HR= 0.59; 95% CI: 0.31-1.11; P=0.100) or freezing all embryos (model 1: HR= 1.15; 95% CI: 0.59-2.24; P=0.685; model 2: HR= 0.94; 95% CI: 0.51-1.73; P=0.839) had the similar CLBR whether underwent fixed or flexible GnRH-ant protocol. Lastly, using propensity score matching in a 1:1 ratio, 83 pairs of women were selected and analyzed (**Supplementary Table 1**), and no significant difference was found in relation to all the baseline characteristics between groups (all P > 0.05), as well as CLBR (26.5% vs 14.5%, P=0.083) and TTLB (11.43 vs 10.66 months, p=0.576). After applying the Cox models in women ≥ 40 years, the study results did not differ (**Supplementary Table 2**).

**Table 4:** Cox proportional hazard models for CLBR.

	Covariate	Estimate	St. Error	Statistic	Hazard ratio (95%CI)	P value
Total population						
Antagonist unadjusted	Fixed GnRH-ant protocol				Reference	
	Flexible GnRH-ant protocol	-0.19	0.20	-0.93	0.83 [0.56, 1.23]	0.351
Antagonist adjusted model 1^a^	Fixed GnRH-ant protocol				Reference	
	Flexible GnRH-ant protocol	-0.16	0.24	-0.65	0.85 [0.53, 1.38]	0.518
Antagonist adjusted model 2^b^	Fixed GnRH-ant protocol				Reference	
	Flexible GnRH-ant protocol	-0.15	0.21	-0.71	0.86 [0.57, 1.30]	0.477
Cycles with fresh ET						
Antagonist unadjusted	Fixed GnRH-ant protocol				Reference	
	Flexible GnRH-ant protocol	-0.34	0.29	-1.17	0.71 [0.40, 1.26]	0.241
Antagonist adjusted model 1^a^	Fixed GnRH-ant protocol				Reference	
	Flexible GnRH-ant protocol	-0.43	0.37	-1.15	0.65 [0.31, 1.35]	0.250
Antagonist adjusted model 2^b^	Fixed GnRH-ant protocol				Reference	
	Flexible GnRH-ant protocol	-0.53	0.32	-1.64	0.59 [0.31, 1.11]	0.100
Freeze-all cycles						
Antagonist unadjusted	Fixed GnRH-ant protocol				Reference	
	Flexible GnRH-ant protocol	-0.26	0.28	-0.91	0.77 [0.44, 1.35]	0.361
Antagonist adjusted model 1^a^	Fixed GnRH-ant protocol				Reference	
	Flexible GnRH-ant protocol	0.14	0.34	0.41	1.15 [0.59, 2.24]	0.685
Antagonist adjusted model 2^b^	Fixed GnRH-ant protocol				Reference	
	Flexible GnRH-ant protocol	-0.06	0.31	-0.20	0.94 [0.51, 1.73]	0.839

a. Model 1 adjusted for age, BMI, duration of infertility, type of infertility, basal FSH level, basal LH level, antral follicle count, and infertility indicators.

b. Model 2 adjusted for all variables in Model 1, with multiple imputation to missing values.

GnRH-ant, gonadotropin releasing hormone antagonist.

## Discussion

To our knowledge, this is the first study to compare the efficacy of fixed GnRH-ant protocol with flexible GnRH-ant protocol in AMA women. In our study, the median age of the women in both groups was 38 years old, which is considered the turning point at which fertility abilities significantly decline (13). Our results suggest that for AMA women, there are no appreciable differences regarding CLBR, TTLB, and other pregnancy outcomes between women undergoing fixed or flexible GnRH-ant protocol.

Although GnRH-ant protocols have become one of the most commonly used regimens in IVF-ET, there is still controversy regarding the optimal GnRH-ant initiation time (14). Two previous systematic reviews compared the efficacy of fixed and flexible GnRH-ant protocols. Al-Inany et al. reported no significant difference between the two protocols, but also emphasized the trend of higher pregnancy rate of fixed GnRH-ant protocol. The latest review by Venetis et al. included more studies and reported that fixed GnRH-ant protocol resulted in a higher ongoing pregnancy rate (3, 10). However, reproductive characteristics vary greatly among different populations, while very few studies exist analyzing the optimal timing of GnRH-ant initiation for specific subpopulations. A randomized controlled trial (RCT) evaluated the number of oocytes retrieved in high ovarian responders and found comparable results of pregnancy outcomes between fixed and flexible GnRH-ant groups (15). Similar studies specifically focusing on AMA women are lacking.

Throughout all comparisons in our study, although fixed GnRH-ant protocol showed a trend of advantage over flexible protocol, the difference was not statistically significant to draw any positive conclusion. To provide a reasonable explanation for our findings, one assumption is the consistent variability of the LH profiles in the two groups. In our study, both groups showed comparable serum LH levels before COS and on the trigger day. In terms of premature LH surge rate, the two strategies showed a similar result (23.1% vs 25.2%, P=0.730), but the women in our study seem more likely to have premature LH surge during COS compared with previous studies (15–17). Numerous studies have reported low serum LH level (18, 19), as well as high serum LH level during COS (9, 19), would impair pregnancy and live birth outcomes. When it comes to the choice of GnRH-ant initiation strategy, according to previous studies, the inhibition effect of GnRH-ant on LH level did not differ between fixed or flexible GnRH-ant protocols, which are in accordance with our results (16, 17).

In our study, we chose CLBR as the primary study outcome, which is presently considered the most objective outcome to reflect the success of an IVF-ET cycle and is also not assessed by previous studies on GnRH-ant initiation strategies (20). Besides, we also introduced TTLB, a time-dependent outcome, to evaluate the required time for women to achieve a live birth, which is extremely important for AMA women with a shorter and precious fertility window (21). Survival analysis methods including Kaplan-Meier curves and Cox proportional hazard model were applied to assess the outcomes. We also conducted various subgroup analysis to assess CLBR within women with fresh embryo transfer or freezing all embryos, and in women aged ≥ 40 years. Propensity score matching was applied to alleviate the confounding factors resulting from imbalanced baseline characteristics. However, there are limitations in our study. First, the reliability of conclusions was interfered with by the retrospective and single-center study design, as well as the limited sample size. Another limitation was the confounding factors in GnRH-ant initiation protocols, which is also a common problem in all previous studies on GnRH-ant initiation strategies. That is, for those who initiated GnRH-ant on day 6 of COS, if they meet the criteria for the flexible GnRH-ant protocol at the same time, it will be not rigorous enough to divide them directly into the fixed group. One possible solution would be to exclude those women who also fulfilled the criteria for flexible protocol when determining the population with the fixed GnRH-ant protocol. However, such screening would cause an unacceptable reduction in sample size for our study.

## Conclusion

Women with AMA may benefit from clinical therapies and strategies like individualized ovarian stimulation, oocyte cryopreservation, and recombinant LH supplementation (6, 22). In our study, different GnRH-ant initiation strategies did not affect long-term reproduction outcomes for AMA women. Therefore, GnRH-ant strategies can be tailored to the clinical situation, and the timing of GnRH-ant initiation can be adjusted flexibly. The conclusion should be interpreted with caution and further RCTs on long-term pregnancy outcomes are necessary to validate.

## Data availability statement

The raw data supporting the conclusions of this article will be made available by the authors, without undue reservation.

## Ethics statement

The studies involving humans were approved by Ethics Committee of the affiliated hospital of SDUTCM. The studies were conducted in accordance with the local legislation and institutional requirements. The ethics committee/institutional review board waived the requirement of written informed consent for participation from the participants or the participants’ legal guardians/next of kin because retrospective nature and anonymous data of this study.

## Author contributions

QH: Conceptualization, Formal analysis, Investigation, Methodology, Software, Visualization, Writing – original draft. YZ: Methodology, Supervision, Writing – original draft. XY: Conceptualization, Investigation, Software, Writing – original draft. KA: Software, Supervision, Validation, Writing – original draft. JS: Conceptualization, Supervision, Validation, Visualization, Writing – review & editing. ZS: Conceptualization, Funding acquisition, Validation, Writing – review & editing.
