# Supplementary material for: Optimal timing of GnRH antagonist initiation in IVF-ET: a retrospective cohort study on advanced maternal age women
Source: Front Endocrinol (Lausanne). 2024 Feb 5;15:1340230. doi: 10.3389/fendo.2024.1340230 (PMC10875460; doi:10.3389/fendo.2024.1340230)
Supplement: Supplementary file 1 [file Table_1.docx]

Supplementary table 1 Baseline characteristics and primary outcomes in the original cohort and cohort after propensity score matching

| **Characteristic** | **Original cohort** | | | **After** **propensity score matching** | | |
| --- | --- | --- | --- | --- | --- | --- |
|  | **Fixed group** | **Flexible group** | **P value** | **Fixed group** | **Flexible group** | **P value** |
| Patients | 136 | 336 |  | 83 | 83 |  |
| Age (years) | 38.00 [36.00, 41.00] | 38.00 [36.00, 41.00] | 0.936 | 38.00 [36.00, 40.50] | 39.00 [37.00, 41.00] | 0.210 |
| BMI (kg/m2) | 23.20 [21.20, 25.00] | 23.65 [21.65, 26.00] | 0.059 | 22.90 [21.20, 24.80] | 22.60 [21.00, 25.00] | 0.913 |
| Duration of infertility | 3.00 [1.00, 5.00] | 3.00 [1.00, 5.00] | 0.757 | 3.00 [2.00, 4.00] | 3.00 [1.00, 5.00] | 0.816 |
| Type of infertility |  |  | 1.000 |  |  | 0.697 |
| Primary infertility | 29 (21.3) | 71 (21.1) |  | 15 (18.1) | 18 (21.7) |  |
| Secondary infertility | 107 (78.7) | 265 (78.9) |  | 68 (81.9) | 65 (78.3) |  |
| Basal FSH level (IU/L) | 7.91 [6.52, 9.81] | 7.72 [6.32, 9.73] | 0.658 | 7.82 [6.38, 9.46] | 7.29 [6.28, 8.94] | 0.366 |
| Basal LH level (IU/L) | 4.18 [3.46, 5.99] | 4.26 [3.09, 5.61] | 0.234 | 4.20 [3.65, 5.81] | 4.33 [3.22, 5.79] | 0.551 |
| Antral follicle count | 16.00 [10.50, 22.50] | 15.00 [10.00, 22.00] | 0.367 | 16.00 [10.00, 21.00] | 15.00 [10.00, 22.00] | 0.710 |
| Infertility indicators |  |  | 0.685 |  |  | 0.807 |
| Female factor | 115 (84.6) | 273 (81.2) |  | 71 (85.5) | 72 (86.7) |  |
| Male factor | 3 (2.2) | 8 (2.4) |  | 2 (2.4) | 3 (3.6) |  |
| Mixed factor | 18 (13.2) | 55 (16.4) |  | 10 (12.0) | 8 (9.6) |  |
| **Primary outcomes** |  |  |  |  |  |  |
| CLBR | 38 (27.9) | 69 (20.5) | 0.105 | 22 (26.5) | 12 (14.5) | 0.083 |
| TTLB (months) | 10.56 [8.73, 12.80] | 10.30 [8.67, 13.23] | 0.782 | 11.43 [9.30, 14.44] | 10.66 [8.98, 13.20] | 0.576 |

Note: Continuous data are represented as median (25th and 75th percentile) because of nonnormal distribution, and categorical variables are represented as number (%). There are no significant difference between the groups either before or after propensity score matching. BMI: Body mass index; FSH: Follicle-stimulating hormone; LH: Luteinizing hormone; CLBR: Cumulative live birth rate; TTLB: Time to first live birth.
