# Supplementary material for: Optimal timing of GnRH antagonist initiation in IVF-ET: a retrospective cohort study on advanced maternal age women
Source: Front Endocrinol (Lausanne). 2024 Feb 5;15:1340230. doi: 10.3389/fendo.2024.1340230 (PMC10875460; doi:10.3389/fendo.2024.1340230)
Supplement: Supplementary file 2 [file Table_2.docx]

Supplementary table 2 Cox proportional hazard models for CLBR in women aged ≥40 years.

|  | **Covariate** | **Estimate** | **St. Error** | **Statistic** | **Hazard ratio (95%CI)** | **P value** |
| --- | --- | --- | --- | --- | --- | --- |
| **Women aged ≥40 years** |  |  |  |  |  |  |
| Antagonist unadjusted | Fixed protocol |  |  |  | Reference |  |
|  | Flexible protocol | -0.32 | 0.58 | -0.55 | 0.72 [0.23, 2.28] | 0.582 |
| Antagonist adjusted model 1^a^ | Fixed protocol |  |  |  | Reference |  |
|  | Flexible protocol | -0.13 | 0.88 | -0.14 | 0.88 [0.16, 4.96] | 0.885 |
| Antagonist adjusted model 2^b^ | Fixed protocol |  |  |  | Reference |  |
|  | Flexible protocol | 0.30 | 0.83 | 0.36 | 1.35 [0.26, 6.94] | 0.716 |

a. Model 1 adjusted for age, BMI, duration of infertility, type of infertility, basal FSH level, basal LH level, antral follicle count, and infertility indicators.

b. Model 2 adjusted for all variables in Model 1, with multiple imputation to missing values.
